# Selenium‐Enriched Submerged Fermentation of *Tricholoma matsutake* and Antioxidant and Hypoglycemic Activities of Its Mycelial Polysaccharide

**DOI:** 10.1002/fsn3.71224

**Published:** 2025-11-23

**Authors:** Jinkun Liu, Yu Wang, Zhili Zhong, Mengli Yu, Xinchao Zhou, Huarong Luo, Yuxin Xiang, Man Xu, Zheli Yi, Xiqiang Zhu, Xiaopeng Liu, Ning Jiang

**Affiliations:** ^1^ Hubei Key Laboratory of Biologic Resources Protection and Utilization Hubei Minzu University Enshi P. R. China; ^2^ School of Biological Science and Technology Hubei Minzu University Enshi P. R. China

**Keywords:** antioxidant, hypoglycemia, selenium enrichment, submerged fermentation, *T. matsutake* polysaccharides

## Abstract

*Tricholoma matsutake* mycelial polysaccharides (TMP) exhibit a variety of biological activities; however, research on the biological activities of selenium‐enriched *T. matsutake* mycelial polysaccharides (Se‐TMP) remains limited. To compare the antioxidant and hypoglycemic activities of TMP and Se‐TMP, the optimal selenium tolerance concentration for the strains and the ideal selenium supplementation for liquid fermentation were determined first. Polysaccharide extraction was scaled up under optimized conditions. The antioxidant activities of TMP and Se‐TMP were evaluated in vitro, and both were administered by gavage to T2DM miceinduced by a high‐fat diet and streptozotocin. The results showed that the maximum selenium tolerance of *T. matsutake* strains was 50 μg/mL Na_2_SeO_3_ on plates. The highest mycelial yield (1.04 ± 0.07 g/100 mL) occurred at 30 μg/mL Na_2_SeO_3_ in liquid culture. Both TMP and Se‐TMP exhibited significant in vitro antioxidant activity. In vivo, both polysaccharides lowered blood glucose, improved glucose and lipid metabolism, reduced lipid levels, increased fasting insulin (FINS), improved HbA1c levels, enhanced hexokinase (HK) and pyruvate kinase (PK) activities, and alleviated oxidative stress. Selenium enrichment further enhanced the antioxidant and hypoglycemic effects. Thus, submerged fermentation to produce Se‐TMP improves the biological activity of TMP.

## Graphical abstract

The maximum selenium tolerance of *T. matsutake* strains on plates was 50 μg/mL Na₂SeO₃, while the highest mycelial yield (1.04 ± 0.07 g/100 mL) was achieved at 30 μg/mL Na₂SeO₃ in liquid culture. Both TMP and Se‐TMP exhibited significant in vitro antioxidant and in vivo hypoglycemic activities in diabetic mice, with Se‐TMP being more effective. Thus, selenium enrichment further enhanced the antioxidant and hypoglycemic properties of the polysaccharide from *T. matsutake* mycelia.

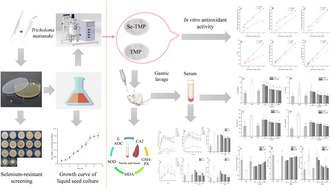

## 1 Introduction

Diabetes mellitus is a chronic metabolic disorder characterized by hyperglycemia, resulting from impaired insulin secretion or insulin resistance. The global incidence of diabetes is steadily increasing, driven by improved living standards and changing dietary habits, making it a major non‐communicable disease that poses a significant threat to human health (Huang et al. 2015). It is estimated that 90% of diabetes patients are diagnosed with type 2 diabetes mellitus (T2DM) after the age of 40. T2DM is also closely associated with complications such as dyslipidemia, diabetic nephropathy, non‐alcoholic fatty liver disease, atherosclerosis, coronary heart disease, and cardiovascular disease (Guo et al. 2020). Currently, a variety of hypoglycemic agents are used to treat T2DM, including metformin, acarbose, sulfonylureas, glinides, α‐glucosidase inhibitors, thiazolidinediones, dipeptidyl peptidase IV inhibitors, sodium‐glucose cotransporter 2 inhibitors, glucagon‐like peptide 1 agonists, and insulin. However, these drugs are associated with various side effects (Fadini and Longato 2024). For example, more than 50% of patients on long‐term metformin experience gastrointestinal distress due to inhibition of bile acid absorption (Wu et al. 2016), while acarbose, which reduces postprandial and fasting blood glucose levels by inhibiting carbohydrate digestion in the small intestine, can cause flatulence, diarrhea, bloating, and nausea (Dong et al. 2022). Given these concerns, there is a growing need to discover novel treatments for diabetes that either have minimal or no toxic side effects. In this regard, dietary polysaccharide interventions have emerged as a promising area of research for the prevention and management of diabetes.

Polysaccharides are macromolecular polymers composed of more than 10 monosaccharide units linked by glycosidic bonds. These compounds offer a range of significant biological benefits with minimal or no toxicity (Tao et al. 2024). As essential biomolecules, polysaccharides play a critical role in the proper functioning of organisms and are involved in various important biological activities. Natural polysaccharides, predominantly derived from the cell walls and membranes of bacteria, plants, and fungi, are known for their low toxicity and minimal side effects (Xue et al. 2023). These complex macromolecules, with multiple biological targets, can exert neuroprotective effects through various mechanisms and play an important role in regulating blood glucose and lipids (Feng and Hao 2024).

Selenium (Se) is an essential trace element known for its antioxidant and anti‐inflammatory properties (Meiran et al. 2023). It plays a crucial role in maintaining a healthy immune system and protecting cells from oxidative stress (Zhu et al. 2019). According to the Dietary Reference Intakes for Chinese Residents (National health and family planning commission 2017), the recommended daily intake of selenium for adults in China is 60 μg, with an estimated average requirement of 50 μg. The tolerable upper intake level is set at 400 μg per day. The average daily selenium intake of Chinese residents is lower than the recommended standard, which may pose multiple hazards to physical health. In nature, selenium primarily exists in the inorganic forms of selenate (SeO₄^2−^), selenite (SeO₃^2−^), and elemental selenium, all of which have a narrow safety margin. However, by combining inorganic selenium with polysaccharides, peptides, or proteins through biotransformation, organic selenium compounds can be created, which significantly improve the safety profile of inorganic selenium (Zhu et al. 2020). Although definitive conclusions about the optimal daily intake of selenium and its relationship to type 2 diabetes mellitus (T2DM) risk are still lacking, most studies suggest that insufficient selenium intake increases the risk of T2DM. Selenium deficiency has also been linked to neurological dysfunction and an elevated risk of cancer (Chen et al. 2013; Robert et al. 2010). Polysaccharides themselves exhibit notable biological activities, which are further enhanced when combined with selenium (Li et al. 2018). The use of selenium‐polysaccharide complexes as nutritional supplements not only optimizes the physiological and pharmacological functions of both components but also addresses their inherent limitations, such as low solubility and bioavailability (Schrauzer 2001).

*Tricholoma matsutake*, a species belonging to the subgenus *Tricholoma*, is a traditional edible and medicinal mushroom native to Asian countries, including China, Japan, and Korea (Ding et al. 2013). It is well known for its potent anti‐inflammatory and immune‐modulating properties, as well as its significant medicinal value. For centuries, *T. matsutake* has been used in the treatment of diabetes and cardiovascular diseases (Hua et al. 2016). In recent years, there has been growing interest in deriving biologically active natural products from medicinal and edible fungi, largely due to their low toxicity and minimal side effects. The polysaccharides from *T. matsutake* (TMP) are considered its main medicinal components, exhibiting strong anti‐inflammatory and antioxidant effects (Ding et al. 2010). While much research has focused on the hypoglycemic and hypolipidemic activities of *T. matsutake* polysaccharides (Yang et al. 2021), studies on the bioactivity of selenium‐enriched *T. matsutake* polysaccharides (Se‐TMP) remain limited.

Submerged fermentation allows for precise control over medium and culture conditions, offering continuous production, a short fermentation time (6–7 days), and high efficiency (Noushahi et al. 2024). As a result, the production of mycelium through submerged fermentation and the extraction of active compounds have become key methods for developing and utilizing rare edible mushrooms. By using edible fungi as a carrier, the addition of inorganic selenium to the liquid medium, followed by its conversion into organic selenium, has emerged as an effective strategy for producing selenium‐enriched products. In this study, we assessed the maximum selenium tolerance of *T. matsutake* strains, optimized the inorganic selenium concentration in the culture medium, and successfully produced large quantities of selenium‐enriched *T. matsutake* mycelium through fermentation. Se‐TMP was then extracted and the in vitro antioxidant and hypoglycemic activities of TMP and Se‐TMP were compared, providing a theoretical basis for the development and application of selenium‐enriched products derived from rare edible mushrooms.

## 2 Materials and Methods

### 2.1 Materials and Reagents

*T. matsutake* strain was purchased from Guangdong Provincial Microbial Culture Preservation Center (GDMCC NO. 5.522), preserved at Hubei Key Laboratory of Biologic Resources Protection and Utilization, Hubei Minzu University, Enshi City, Hubei Province, China. Stock cultures were maintained on potato dextrose agar (PDA) slants, incubated at 25°C for 8 days, and then stored at 4°C. Subcultures were made at 3‐month intervals for subsequent experiments.

Agar powder was obtained from Obstar Biocomputing Co. Ltd. (Beijing, China). Soluble starch was purchased from Tianli Chemical Reagent Co. Ltd. (Tianjin, China). Dipotassium hydrogen phosphate was supplied by Shanghai Test Laboratory Equipment Co. Ltd. (Shanghai, China), while magnesium sulfate was obtained from Bodhi Chemical Co. Ltd. (Tianjin, China). Vitamin B1 (VB1), 1,1‐diphenyl‐2‐picrylhydrazyl (DPPH), streptozotocin (STZ), and 2,2‐biazobis‐(3‐ethylbenzothiazoline‐6‐sulfonate) (ABTS) were purchased from Source Leaf Biotech Co. Ltd. (Shanghai, China). Soybean powder was acquired from Wuhan Foods Co. Ltd., and sodium citrate was obtained from Asia‐Pacific United Chemical Co. Ltd. (Wuxi, China). Citric acid was supplied by Dingshengxin Chemical Co. Ltd. (Tianjin, China). Glucose, vitamin C (Vc), metformin, hydrogen peroxide (H_2_O_2_), and anhydrous ethanol were purchased from Sinopharm Chemical Reagent Co. Ltd. Ferrozine was obtained from Bioengineering Co. Ltd. (Wuhan, China). Sodium acetate, potassium hexacyanoferrate, and ferrous sulfate were supplied by Tianjin Guangfu Science and Technology Development Co. Ltd. Sodium selenite, ferric chloride, and trichloroacetic acid were purchased from West Asia Chemical Technology Co. Ltd. (Shandong, China). Disodium ethylenediaminetetraacetic acid (EDTA‐2Na) was obtained from Fuchen Chemical Reagent Co. Ltd. (Tianjin, China). The assay kits were purchased from Jiancheng Bioengineering Research Institute (Nanjing, China), and all other reagents used were of analytical purity.

### 2.2 Methods

#### 2.2.1 Medium Formulation

The solid medium used was potato dextrose agar (PDA); the liquid seed medium was composed of 200 g potato, 20 g glucose, 1.5 g MgSO_4_, 1 g K_2_HPO_4_, and 0.008 g vitamin B_1_, which were dissolved in 1 L of distilled water, with the pH adjusted to natural levels; the submerged fermentation medium contained 2.1% (m/v) corn starch, 3.9% (m/v) soybean flour, 0.025% (m/v) KH_2_PO_4_, 0.025% (m/v) MgSO_4_, and 0.004% (m/v) vitamin B_6_.

#### 2.2.2 Screening of Selenium Tolerance in *T. Matsutake* Mycelium

The strains were inoculated onto PDA medium and incubated at 25°C. Once the mycelium covered the entire surface of the PDA, 1 cm diameter sections of actively growing mycelium were transferred to PDA medium containing Na₂SeO₃ at concentrations of 0, 2, 4, 6, 8, 10, 20, 30, 40, 50, 60, 70, 80, 90, 100, 200, and 250 μg/mL. The cultures were incubated at 25°C for 22 days in the inverted position. Mycelial growth, morphology, and color were observed to determine the selenium tolerance concentration.

#### 2.2.3 Determination of Liquid Seed Culture Time

Active mycelium (1 cm in diameter) from the PDA medium was transferred to the liquid seed medium and incubated at 23°C with shaking at 150 rpm. Samples were collected every 24 h, with 3 bottles sampled at each time point. The seed liquid was filtered through a 60‐mesh sieve, and any mycelial impurities were rinsed with distilled water. The mycelium was then dried at 105°C to a constant weight. A growth curve was plotted with incubation time on the *x*‐axis and dry mycelial weight on the *y*‐axis. The optimal incubation time for the liquid seed culture was determined from this growth curve.

#### 2.2.4 Shake Flask Fermentation

A 10% inoculum of *T. matsutake* liquid culture was transferred to a 250 mL conical flask containing 100 mL of fermentation medium. The flask was incubated at 23°C with shaking at 150 rpm for 7 days.

#### 2.2.5 Determination of Optimum Selenium Concentration for Liquid Culture

Based on the results of the preliminary experiment, the seed liquid was inoculated into fermentation medium containing Na₂SeO₃ at concentrations of 0, 20, 25, 30, 35, 40, 45, and 50 μg/mL. The cultures were incubated for 7 days, and the dry weight of the mycelium and selenium content was measured.

#### 2.2.6 Determination of Selenium Content

The selenium content was determined using an AFS‐930 atomic fluorescence spectrophotometer manufactured by Beijing Jitian Instrument Co. Ltd., following the method outlined in GB5009.93‐2017 “Determination of Selenium in Food.” Both total selenium and inorganic selenium were quantified, with organic selenium calculated as the difference between total and inorganic selenium content.

#### 2.2.7 Scale‐Up of Selenium‐Enriched and Non‐Selenium‐Enriched *T. Matsutake* Mycelia

A 35 L submerged fermentation medium was prepared and transferred to a 50 L fermenter, then sterilized at 121°C for 30 min. After cooling, a sterilized Na₂SeO₃ solution was added to achieve a final selenium concentration of 30 μg/mL, and the culture was inoculated with a 10% inoculum. Fermentation was conducted under the following conditions: temperature 23°C, dissolved oxygen 20%–80%, and incubation for 6 days. After fermentation, the broth was filtered through a 60‐mesh sieve, and the mycelium was washed with distilled water to remove impurities, then freeze‐dried. A parallel fermentation, without the addition of Na₂SeO₃, was performed under identical conditions to obtain non‐selenium‐enriched *T. matsutake* mycelium.

#### 2.2.8 Extraction of Polysaccharides

Powdered selenium‐enriched and non‐enriched *T. matsutake* mycelium was ultrasonicated at a solid‐to‐liquid ratio of 1:15 (g/mL) for 8 min at 350 W. The extraction continued in a water bath at 90°C for 2 h, followed by centrifugation at 2100 × g for 15 min. The supernatant was concentrated using a rotary evaporator at 68°C under reduced pressure. To the concentrated supernatant, 95% ethanol was added to achieve a final ethanol concentration of 80%, and the mixture was stored overnight at 4°C. The precipitate was collected by centrifugation, redissolved in water, and treated with Sevag reagent to remove free proteins. The solution was then re‐precipitated by adding ethanol, stored at 4°C overnight, and lyophilized to yield the polysaccharide fractions TMP (from non‐enriched mycelium) and Se‐TMP (from selenium‐enriched mycelium).

#### 2.2.9 Antioxidant Activity of TMP and Se‐TMP

##### 2.2.9.1 DPPH Radical Scavenging Activity

The DPPH radical scavenging activity of TMP and Se‐TMP was measured with slight modifications to a standard method (Xiao et al. 2020). Various concentrations of TMP and Se‐TMP were prepared, and 100 μL of 0.1 mmol/L DPPH solution was added to 100 μL of each test solution in a 96‐well plate. The mixture was vortexed briefly and incubated at room temperature for 30 min in the dark. Absorbance was measured at 517 nm, and the DPPH radical scavenging activity was calculated using the following formula:

(1) $$\text{Scavenging rate}\;\left(\%\right)=\left(1-\frac{A1-A3}{A2}\right)\times 100\%$$

where *A2* is the absorbance of the control, *A1* is the is the absorbance of the sample, and *A3* is the absorbance of the blank.

The DPPH radical scavenging activity of the positive control, ascorbic acid (Vc), was measured following the same method. A linear equation for Vc, *y* = 1.532*x* + 14.217 (*R* ^2^ = 0.996), was obtained, where concentration was the independent variable and radical scavenging rate was the dependent variable. Based on this equation, the DPPH scavenging activity of TMP and Se‐TMP was calculated and expressed as Vc equivalents (Yang et al. 2017).

##### 2.2.9.2 ABTS Radical Scavenging Activity

The ABTS radical scavenging activity of TMP and Se‐TMP was determined with minor modifications to the method described in the literature (Gowd et al. 2018). Various concentrations of TMP and Se‐TMP were prepared, and 100 μL of each test solution was added to 300 μL of ABTS working solution in a 96‐well plate. The mixture was shaken and allowed to react at room temperature for 10 min. Absorbance was then measured at 734 nm, and the ABTS radical scavenging activity was calculated using the following formula:

(2) $$\text{Scavenging rate}\;\left(\%\right)=\left(\frac{A0-\mathrm{At}}{A0}\right)\times 100\%$$

where *A0* is Absorbance of control, and *At* is Absorbance of sample.

The ABTS radical scavenging activity of the positive control, ascorbic acid (Vc), was measured in the same manner, yielding a linear equation of *y* = 0.658*x* − 1.411 (*R* ^2^ = 0.999), with concentration as the independent variable and radical scavenging rate as the dependent variable. Based on this equation, the ABTS scavenging activity of TMP and Se‐TMP was calculated and expressed as Vc equivalents.

##### 2.2.9.3 Hydroxyl Radical Scavenging Ability

The hydroxyl radical scavenging activity of TMP and Se‐TMP was measured with slight modifications to the method described in the literature (Chang et al. 2024). In a 96‐well plate, 40 μL of FeSO₄ solution, 80 μL of sodium salicylate, 80 μL of test solution at varying concentrations, and 40 μL of H₂O₂ were mixed. The mixture was shaken and allowed to react at room temperature for 10 min. Absorbance was measured at 510 nm, and the scavenging ability was calculated using the following formula:

(3) $$\text{Scavenging rate}\;\left(\%\right)=\left(1-\frac{\mathrm{As}-\mathrm{Ar}}{A0}\right)\times 100\%$$

where *A0* is the absorbance of the blank, *As* is absorbance of sample, and *Ar* is absorbance of control.

The hydroxyl radical scavenging activity of the positive control, ascorbic acid (Vc), was measured in the same way. A linear equation for Vc was obtained: *y* = 28.031*x* − 1.416 (*R* ^2^ = 0.986), where concentration is the independent variable and scavenging rate is the dependent variable. Based on this equation, the hydroxyl radical scavenging activity of TMP and Se‐TMP was calculated and expressed as Vc equivalents.

##### 2.2.9.4 Ferric‐Reducing Antioxidant Power (FRAP) Assay

The FRAP of TMP and Se‐TMP was detected according to the method described in the literature (Hu et al. 2016) with a slight modification. Different concentrations of TMP and Se‐TMP solutions were prepared, and 40 μL of each test solution was mixed with 200 μL of Fe^3+^‐TPTZ solution. The mixtures were incubated at 37°C for 30 min, and absorbance was measured at 593 nm.

The FRAP of the positive control, ascorbic acid (Vc), was determined following the same procedure. A linear equation for Vc was obtained: *y* = 0.0045*x* + 0.1023 (*R* ^2^ = 0.999), where concentration is the independent variable and FRAP is the dependent variable. Based on this equation, the FRAP of TMP and Se‐TMP was calculated and expressed as Vc equivalents.

##### 2.2.9.5 Determination of Fe^2+^ Chelating Capacity

The ferrous ion chelating ability of TMP and Se‐TMP was determined following a method from the literature (Zhang et al. 2017) with slight modifications. TMP and Se‐TMP were prepared at different concentrations, and 50 μL of each test solution was mixed with 5 μL of ferrous chloride solution and 160 μL of deionized water. The mixture was shaken at room temperature for 5 min, followed by the addition of 10 μL of ferrozine solution. The reaction was allowed to proceed at room temperature for 10 min. Absorbance was measured at 562 nm, and the chelating capacity was calculated using the formula:

(4) $$\text{Chelating capacity}\left(\%\right)=\left(1-\frac{A1-A2}{A0}\right)\times 100\%$$

where *A0* is the absorbance of blank; *A1* is the absorbance of sample, and *A2* is the absorbance without ferrozine.

The chelating ability of the positive control, EDTA‐2Na, was measured in the same manner. A linear equation for the chelating capacity of EDTA‐2Na was obtained: *y* = 0.0036*x* + 0.1108 (*R* ^2^ = 0.992). The chelating capacity of TMP and Se‐TMP was calculated based on this equation and expressed as EDTA‐2Na equivalents.

##### 2.2.9.6 Total Reducing Power

The total reducing power of TMP and Se‐TMP was measured using a modified version of the method described in the literature (Zhang et al. 2017). The reducing power of the positive control, ascorbic acid (Vc), was determined, and a linear equation for the reducing power was obtained: *y* = 0.0015*x* + 0.0086 (*R* ^2^ = 0.995), where the concentration was the independent variable and absorbance was the dependent variable. Based on this equation, the total reducing power of TMP and Se‐TMP was calculated and expressed as Vc equivalents.

#### 2.2.10 Hypoglycemic Activity of Se‐TMP and TMP In Vivo

##### 2.2.10.1 Animals

Male ICR mice (20 ± 2 g) were used in the animal model experiment and purchased from Changsheng Biotechnology Co. Ltd. (Liaoning, China) (License number: No. SCXK (Liao) 2020‐0001). The mice were housed in an animal facility maintained at a temperature of 23°C ± 2°C, with a 12/12‐h light–dark cycle and relative humidity of 65%–70%. They had ad libitum access to food and water. The animal studies were approved by the Hubei Minzu University Animal Ethics Committee, Enshi, China, and all experimental procedures were conducted in accordance with the university's Guidelines for Animal Experimentation.

##### 2.2.10.2 Experimental Design

After 7 days of acclimatization, 90 mice were randomly assigned to 9 groups (*n* = 10 per group): Normal Control (NC), Diabetes Model (DM), Diabetes Positive Control (PC), Se‐TMP low, medium, and high‐dose groups (Se‐TMP‐L, Se‐TMP‐M, Se‐TMP‐H), and TMP low, medium, and high‐dose groups (TMP‐L, TMP‐M, TMP‐H).

##### 2.2.10.3 Streptozotocin‐Induced Diabetic Mice

All mice, except those in the NC group, were intraperitoneally injected with a freshly prepared 1% streptozotocin (STZ) solution (50 mg/kg) for 4 consecutive days Fasting blood glucose levels were measured 3 days after the last injection (Zhan et al. 2015). Mice with fasting blood glucose levels ≥ 11.1 mmol/L were considered diabetic and included in subsequent experiments.

##### 2.2.10.4 Treatment

Mice in the NC and DM groups were gavaged with sterile water. Mice in the PC group received metformin hydrochloride (200 mg/kg/day) via gavage. Mice in the Se‐TMP‐L, Se‐TMP‐M, and Se‐TMP‐H groups were gavaged with Se‐TMP at doses of 50, 100, and 200 mg/kg/day, respectively. Mice in the TMP‐L, TMP‐M, and TMP‐H groups were gavaged with TMP at 50, 100, and 200 mg/kg/day. The dose of TMP and Se‐TMP was determined based on previous experiments (Zhai et al. 2013). During the treatment period, mice in the NC group were fed a standard diet, while mice in the other groups were fed a high‐fat diet. Treatments were administered once daily for 7 weeks, and blood glucose levels were measured weekly.

##### 2.2.10.5 Physiological and Biochemical Analysis

During the treatment period, fasting blood glucose levels were monitored and recorded weekly. At the end of treatment, mice were fasted for 8 h and subjected to an oral glucose tolerance test (OGTT). All mice were gavaged with glucose (2 g/kg), and blood was collected from the orbital venous plexus at 0, 30, 60, 90, and 120 min to measure blood glucose levels. The blood glucose concentration over time was plotted, and the area under the curve (AUC) was calculated (Xu et al. 2021). After the OGTT, mice were fasted for an additional 8 h and then underwent an oral fat tolerance test (OFTT). Each group was gavaged with olive oil (10 mL/kg), and serum triglyceride (TG) levels were measured at 0, 1, 2, 3, and 4 h. The TG concentration over time was plotted, and the AUC was calculated (Mbagwu et al. 2020).

At the end of the experiment, whole blood was collected and serum was separated immediately (2100 × g, 4°C, 10 min). Biochemical indices, including total cholesterol (TC), triglycerides (TG), low‐density lipoprotein cholesterol (LDL‐C), free fatty acids (FFA), fasting insulin (FINS), and glycosylated hemoglobin (HbA1c), were measured. Mouse livers were washed with saline, weighed, and minced. A 10% liver homogenate was prepared by adding 9 volumes of saline, followed by centrifugation at 1000 × g for 10 min. The supernatant was used to measure the activities of hexokinase (HK) and pyruvate kinase (PK). Additionally, the activities of superoxide dismutase (SOD), catalase (CAT), total antioxidant capacity (TAC), glutathione peroxidase (GSH‐Px), and malondialdehyde (MDA) levels were measured in both serum and liver samples.

### 2.3 Statistical Analysis

Data are presented as mean ± standard deviation and were analyzed using Origin 2019 and SPSS Statistics 26 software. Multiple comparisons were performed using the least significant difference (LSD) method. Statistical significance was set at *p* < 0.05. All experiments were repeated three times.

## 3 Results and Discussion

### 3.1 Determination of Selenium‐Tolerant Concentration in *T. Matsutake* Mycelium

**FIGURE 1:**
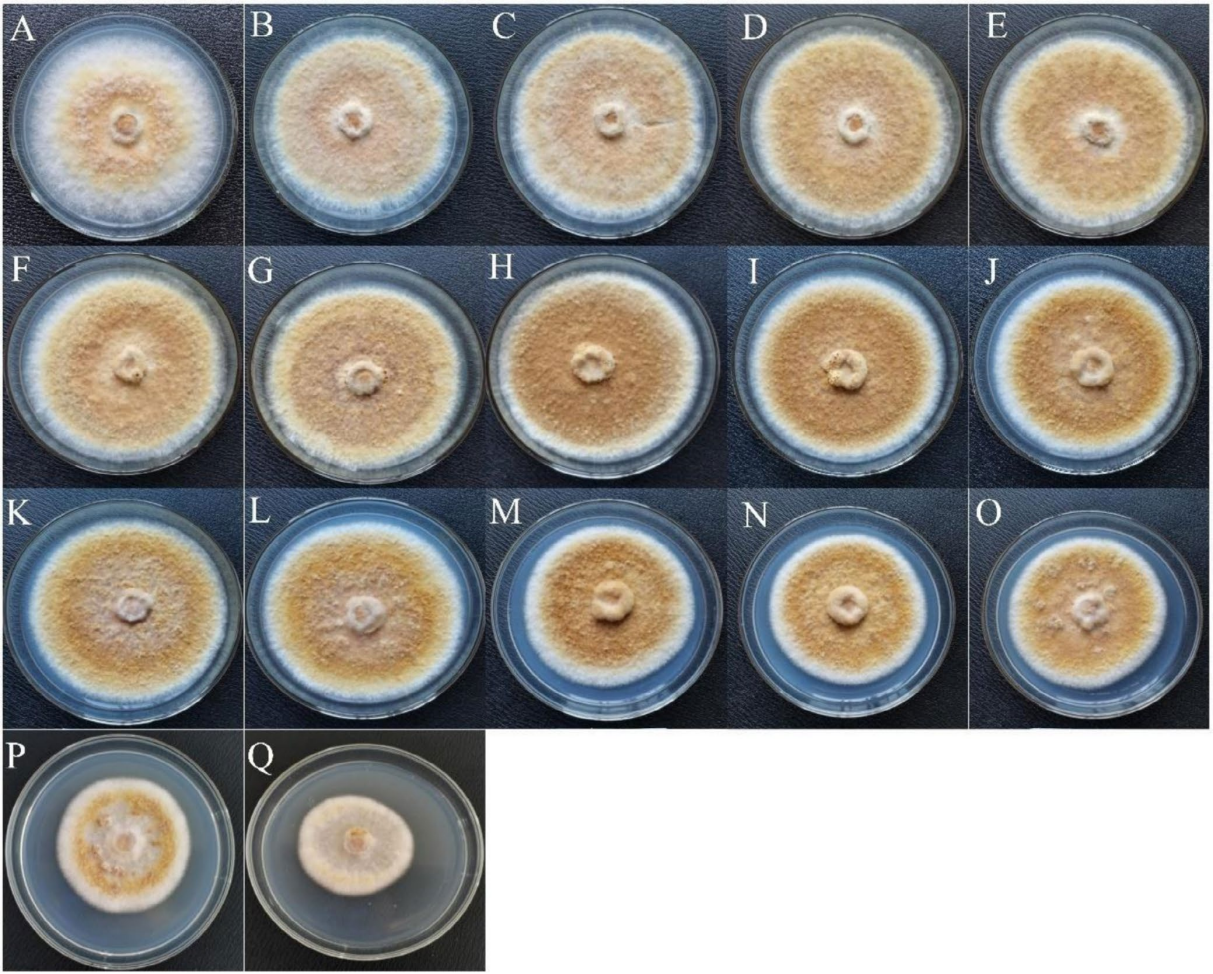
Effect of selenium concentration on the growth of *T. matsutake*. The Na_2_SeO_3_ concentrations for panels (A–Q) were 0, 2, 4,6, 8, 10, 20, 30, 40, 50, 60, 70, 80, 90, 100, 200, and 250 μg/mL.

The growth of *T. matsutake* mycelium on plates containing Na_2_SeO_3_ at concentrations ranging from 0 to 250 μg/mL is shown in Figure 1. In the absence of Na_2_SeO_3_ (Figure 1A), the mycelium grew vigorously with long aerial hyphae. At a Na_2_SeO_3_ concentration of 20 μg/mL (Figure 1G), the mycelium grew even more vigorously, with longer aerial hyphae, indicating that low selenium concentrations promoted *T. matsutake* growth. However, as the selenium concentration increased, browning of the mycelium was observed, although growth was not significantly inhibited. The aerial mycelium growth was notably weakened (Figure 1J,K). At Na_2_SeO_3_ concentrations of 100 μg/mL or higher, the growth of *T. matsutake* mycelium was clearly inhibited (Figure 1O–Q).

The growth behavior of mycelia from different edible fungi on plates containing Na₂SeO₃ varied. For example, *Pleurotus ostreatus* showed significant growth inhibitionat Na₂SeO₃ concentrations above 20 μg/mL (Milovanović et al. 2014), while *Morchella esculenta* grew well at a concentration of 20 μg/mL (Qian et al. 2023). In contrast, *T. matsutake* mycelium maintained robust growth even at Na₂SeO₃ concentrations up to 50 μg/mL. These results indicate that *T. matsutake* exhibits strong tolerance to selenium, making it an ideal candidate for selenium enrichment studies.

### 3.2 Preparation of Liquid Seeds of *T. matsutake* and Determination of Growth Curve

**FIGURE 2:**
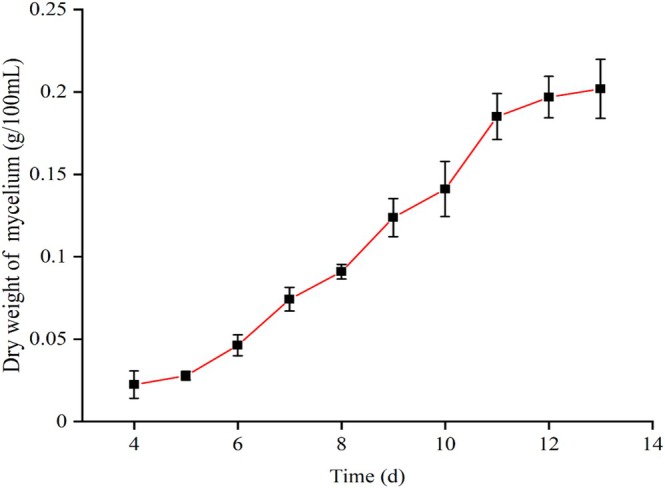
Growth curve of *T. matsutake* liquid seed culture. Each value represents the mean of three measurements (*n* = 3) ± standard deviation.

The growth curve of *T. matsutake* liquid seed culture was plotted with incubation time on the *x*‐axis and the dry weight of *T. matsutake* mycelium on the *y*‐axis (Figure 2). Due to the lack of discernible growth during the initial three‐day lag phase of *T. matsutake* liquid seed culture, measurements of mycelial dry weight were commenced on day 4. The growth curve exhibited an S‐shape, with the culture entering the logarithmic growth phase on day 6 and reaching the plateau phase by day 12. The liquid seed culture on day 9, during the logarithmic growth phase, was selected for subsequent fermentation. The logarithmic phase was chosen for mycelium harvesting because the mycelium grows vigorously at this stage, adapts rapidly to the fermentation environment, and is more likely to yield higher productivity in a short time (Gao et al. 2018).

### 3.3 Determination of the Optimum Selenium Concentration

The effects of Na₂SeO₃ concentration on mycelial dry weight and organic selenium content during subsequent fermentation of *T. matsutake* are shown in Table 1. The selenium tolerance test in shake flasks was conducted to determine the maximum selenium concentration tolerated by *T. matsutake* mycelium in liquid culture, as assessed by biomass yield. However, due to substantial differences in fermentation conditions between shake flasks and bioreactors, the selenium content (both organic and total) measured in shake flask‐derived mycelium should be regarded as preliminary. The definitive quantification of selenium accumulation was established using mycelium from fermenter cultures. Consequently, selenium content in shake flask samples was analyzed only once for reference. At a selenium concentration of 30 μg/mL, the mycelial dry weight reached its maximum of (1.04 ± 0.07) g/100 mL, which was significantly higher than that in the other experimental groups. This concentration also resulted in optimal selenium enrichment. However, increasing the selenium concentration to 50 μg/mL inhibited mycelial growth. This pattern, where selenium promotes growth at lower concentrations but inhibits it at higher concentrations, has also been observed in other edible mushrooms. For instance, the growth of *Lentinula edodes* was inhibited at a Na₂SeO₃ concentration of 69 μg/mL (Kałucka et al. 2023).

**TABLE 1:** Effect of selenium concentration on the dry weight and organic selenium content of *T. matsutake* mycelium.

Concentration of Na₂SeO₃ in medium (μg/mL)	Dry weight of mycelium (g/100 mL)	Total selenium (mg/kg)	Organic selenium (mg/kg)
0	0.83 ± 0.01ABb	4.98	4.71
20	0.83 ± 0.01ABb	365	357
25	0.84 ± 0.15ABb	687	678
30	1.04 ± 0.07Aa	1309	1292
35	0.84 ± 0.05ABb	594	583
40	0.82 ± 0.03ABb	410	400
45	0.81 ± 0.03ABb	311	300
50	0.74 ± 0.03Bb	389	371

*Note:* Upper and lower case English letters are different at the α = 0.01, 0.05 level (*n* = 3), respectively.

### 3.4 Results of Subsequent Fermentation in Fermenter

Following scale‐up fermentation in a bioreactor using the optimized medium, the mycelium of *T. matsutake* achieved a dry weight of 1.36 g/100 mL. The total selenium content reached 336.4 mg/kg, of which 304.7 mg/kg was present in the organic form. It is reported that, *T. matsutake* mycelial remained 1.79 g/100 mL at the 14th day of culture for the *T. matsutake* liquid culture at uncontrolled pH (Kim et al. 2010). This difference was mainly due to the inhibitory effect of high selenium concentrations on subsequent fermentation in our study. The mycelial yield in the fermenter exceeded the theoretical yield obtained from shake flask optimization, likely due to differences in volumetric oxygen transfer coefficients (KLa), dissolved oxygen, and CO_2_ concentrations between the fermenter and shake flask. These findings suggest that the fermenter not only provides a higher yield under the same volume but also offers advantages in large‐scale fermentation, time efficiency, and labor savings. Therefore, the fermenter is the optimal method for large‐scale mycelium cultivation.

### 3.5 Mycelial Polysaccharide

The yield of polysaccharides was 2.69%, with the selenium content of the Se‐TMP solution at 173.5 mg/kg and the organic selenium content at 157 mg/kg. Based on the preparation methods of TMP and Se‐TMP, it is speculated that these two substances primarily consist of polysaccharides, with minor amounts of proteins, nucleic acids, pigments, and minerals. The specific proportions of each component warrant further investigation. In a similar study, selenium enrichment of the edible medicinal mushroom *Antrodia camphorata* through submerged fermentation resulted in a polysaccharide organic selenium content of 66.3 ± 3.9 mg/kg (Li et al. 2023). These results suggest that Se‐TMP is rich in organic selenium and serves as an effective selenium‐enriched material.

### 3.6 In Vitro Antioxidant Activity and Analysis

**FIGURE 3:**
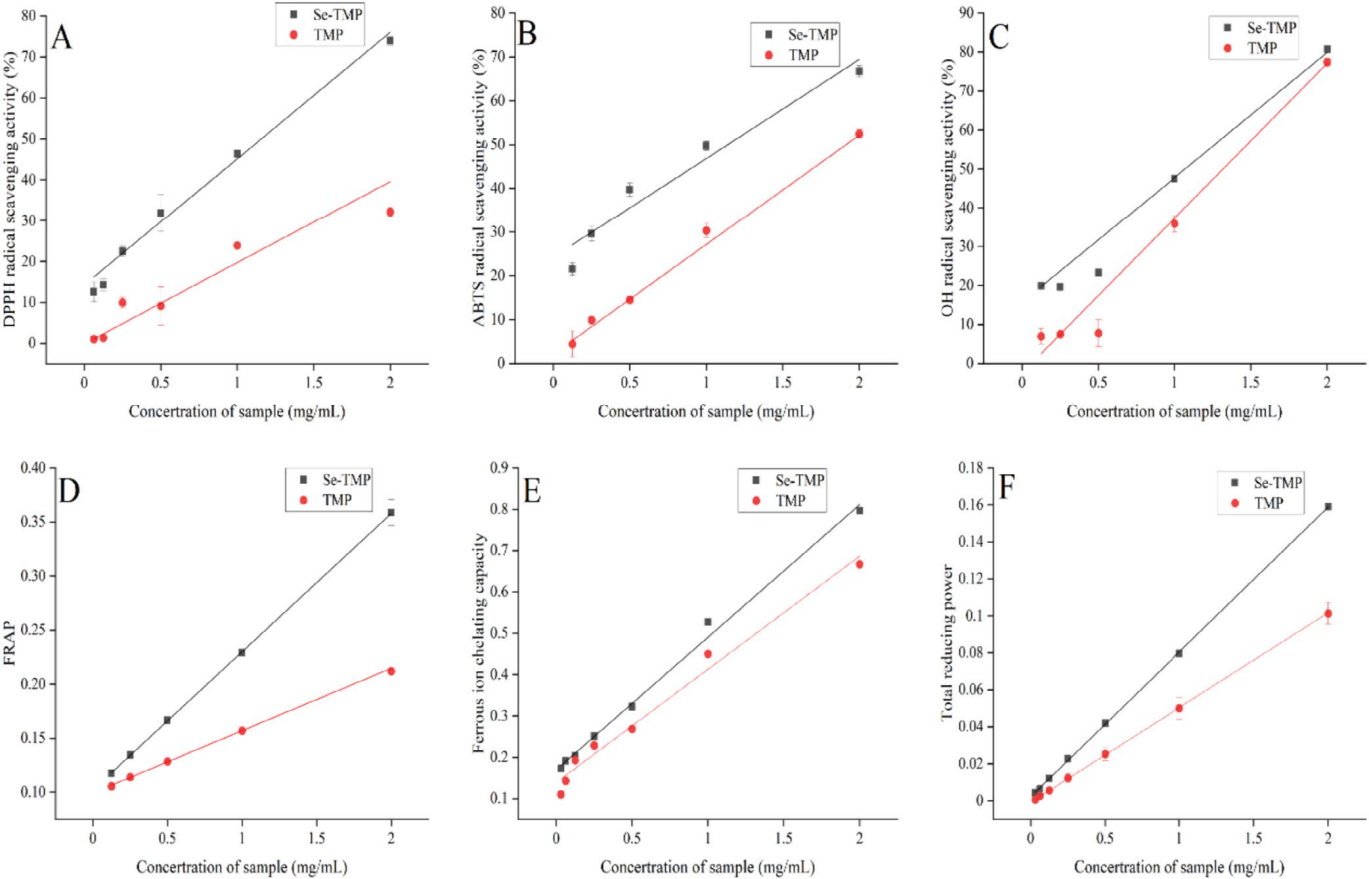
Antioxidant capacities of Se‐TMP and TMP in vitro. Panels (A–F) show the standard curves for DPPH, ABTS, hydroxyl radical scavenging, ferric ion reduction, ferrous ion chelation, and total reducing power, respectively. Each value represents the mean of three measurements (*n* = 3) ± standard deviation.

Numerous studies have reported the antioxidant potential of polysaccharides. In this study, we evaluated the DPPH radical scavenging activity of TMP and Se‐TMP. Se‐TMP demonstrated a stronger radical scavenging capacity than TMP (Figure 3A). The linear relationships between the concentrations of TMP and Se‐TMP (0.0625–2 mg/mL) and their DPPH radical scavenging rates were as follows: TMP: *y* = 19.128*x* + 1.511, *R* ^2^ = 0.952; Se‐TMP: *y* = 31.341*x* + 13.007, *R* ^2^ = 0.988. The DPPH radical scavenging capacities were calculated to be (6.57 ± 0.02) μmolVc equivalents/mg for TMP and (21.77 ± 0.43) μmolVc equivalents/mg for Se‐TMP.

The concentrations of TMP and Se‐TMP (0.125–2 mg/mL) showed a strong linear correlation with their ABTS radical scavenging activities (Figure 3B). The regression equations were: TMP: *y* = 25.157*x* + 2.0025, *R* ^2^ = 0.998, Se‐TMP: *y* = 23.751*x* + 21.72, *R* ^2^ = 0.988. The ABTS radical scavenging capacities were calculated to be (48.22 ± 2.44) μmolVc equivalents/mg for TMP and (76.09 ± 1.56) μmolVc equivalents/mg for Se‐TMP.

Hydroxyl radicals are highly reactive and damaging species that can interact with cellular components, especially DNA, potentially causing mutations or cell death. As shown in Figure 3C, TMP and Se‐TMP both demonstrated strong hydroxyl radical scavenging activity. In the concentration range of 0.125–2 mg/mL, both compounds exhibited a good linear relationship with the hydroxyl radical scavenging rate, with no significant difference in activity. The linear equations were: TMP: *y* = 38.892*x* − 0.456, *R* ^2^ = 0.996, with a scavenging capacity of (1.32 ± 0.02) mmol Vc equivalents/mg. Se‐TMP: *y* = 34.261*x* + 11.784, *R* ^2^ = 0.985, with a scavenging capacity of (1.66 ± 0.17) mmol Vc equivalents/mg. Se‐TMP showed a stronger hydroxyl radical scavenging activity than TMP and Vc.

The ferric reducing antioxidant power (FRAP) assay is commonly used to assess the antioxidant capacity of polysaccharides (Xu et al. 2008). Both TMP and Se‐TMP demonstrated a strong linear relationship with absorbance in the concentration range of 0.125–2 mg/mL (Figure 3D). The linear equation for TMP was *y* = 0.0565*x* − 0.0044, *R* ^2^ = 0.999, and its FRAP value was calculated as (12.04 ± 0.19) μmolVc equivalent/mg. For Se‐TMP, the linear equation was *y* = 0.1281*x* − 0.0001, *R* ^2^ = 0.999, and its FRAP value was (25.85 ± 3.60) μmolVc equivalent/mg.

The ferrous ion chelating ability of TMP and Se‐TMP is shown in Figure 3E. Both compounds exhibited a linear relationship with ferrous ion chelation, with the regression equation for Se‐TMP being *y* = 0.3205*x* + 0.17, *R* ^2^ = 0.995, and for TMP, it was *y* = 0.2737*x* + 0.1392, *R* ^2^ = 0.982. The calculated ferrous ion chelating capacities were (111.46 ± 3.53) μmol EDTA‐2Na equivalent/mg for Se‐TMP and (91.4 ± 2.77) μmol EDTA‐2Na equivalent/mg for TMP.

The relationship between the concentration of Se‐TMP and TMP and their total reducing power is shown in Figure 3F. Both compounds demonstrated a significant linear correlation with reducing power. The regression equation for Se‐TMP was *y* = 0.0784*x* + 0.0021, with *R* ^2^ = 0.999, and for TMP, the equation was *y* = 0.0506*x* − 0.0006, with *R* ^2^ = 0.999. The calculated total reducing powers were (78.94 ± 2.89) μmolVc equivalents/mg for Se‐TMP and (27.96 ± 24.21) μmolVc equivalents/mg for TMP.

The metabolic processes of life generate various free radicals, and the accumulation of these radicals can cause oxidative damage, contributing to aging, cancer, and other chronic diseases. In this study, six antioxidant indices were evaluated to assess whether TMP and Se‐TMP could serve as natural antioxidants. The results showed that, within a certain concentration range, both TMP and Se‐TMP increased the scavenging activity against DPPH and ABTS radicals, hydroxyl radicals, and exhibited higher total reducing power. These findings align with the results reported by Soares et al. for TMP (Soares et al. 2009). TMP also demonstrated strong antioxidant activity in ferrous ion reduction and ferrous ion chelation assays. Notably, Se‐TMP outperformed TMP in most antioxidant indices, particularly in DPPH and ABTS radical scavenging, ferrous ion reduction, and total reducing power. This improvement stems from fundamental differences in their antioxidant mechanisms. Conventional polysaccharides primarily depend on hydrogen atom transfer or single electron transfer from native functional groups (e.g., hydroxyls) to neutralize free radicals—a mechanism that is inherently limited in both versatility and efficiency. In contrast, selenium incorporation into the polysaccharide backbone via chemical bonding introduces distinct antioxidant motifs, notably Se = O and O–Se–O groups (Huang et al. 2020). These selenium‐active sites not only facilitate more efficient electron transfer but also enable multiple reaction pathways—such as the catalytic cycling of selenium between redox states—thereby significantly amplifying the radical quenching capacity. The markedly superior performance of these selenium‐containing groups over conventional hydroxyl‐based scavenging underscores the mechanistic advantage of selenylation. These results suggest that Se‐TMP has superior antioxidant potential, warranting further investigation to explore its potential applications in various industrial and therapeutic contexts.

### 3.7 Effects of TMP and Se‐TMP on Diabetic Mice

#### 3.7.1 Effects of TMP and Se‐TMP on Fasting Blood Glucose, OGTT, OFTT, and FINS Levels in Diabetic Mice

Impaired fasting blood glucose (FBG), glucose tolerance, and fat tolerance are key characteristics of type 2 diabetes mellitus (T2DM), with insulin playing a critical role in regulating glucose and lipid metabolism.

**FIGURE 4:**
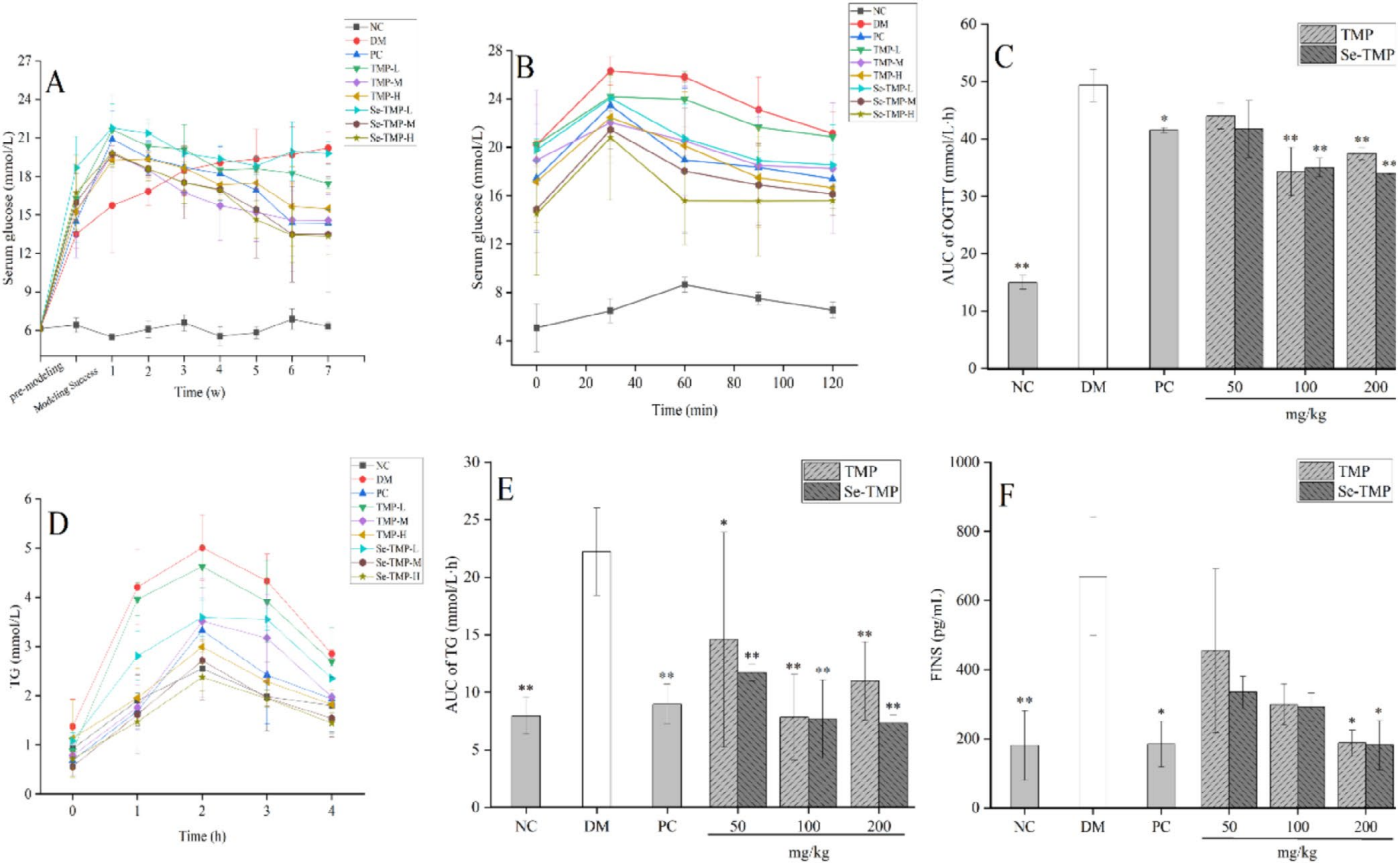
Effect of TMP and Se‐TMP on fasting blood glucose, OGTT, OFTT, and FINS levels. (A) Changes in blood glucose levels in diabetic mice during treatment. (B) Effect of TMP and Se‐TMP on the OGTT. (C) Area under the curve (AUC) for OGTT blood glucose. (D) Effect of TMP and Se‐TMP on the OFTT. (E) Area under the curve (AUC) for OFTT lipid levels. (F) Effects of TMP and Se‐TMP on FINS. Values expressed as mean ± SD (*n* = 10/group). * and ** indicate *p* < 0.05 and *p* < 0.01 compared with the DM group, respectively.

Analysis of FBG levels revealed a rapid increase in STZ‐induced diabetic mice. However, FBG levels began to decrease 2 weeks after treatment, with a more pronounced reduction in the Se‐TMP group compared to the TMP group at the same dose. This decrease stabilized after 6 weeks of treatment (Figure 4A). Effective blood glucose control is essential for preventing or reversing diabetic complications and improving the quality of life in both type 1 and type 2 diabetic patients (Gong et al. 2009). Previous studies have demonstrated the hypoglycemic effects of polysaccharides. For example, *Fructus Corni* polysaccharides significantly reduced FBG levels in diabetic rats (Fu et al. 2021); *Botryosphaeran* polysaccharide effectively lowered glucose levels in diabetic mice (Miranda et al. 2007); *Gynura divaricata* (L.) DC polysaccharide regulates blood glucose by modulating the expression of genes related to PI3K/Akt, AMPK and GS/GSK‐3β signaling pathways (Zhu et al. 2024).

The oral glucose tolerance test (OGTT) is commonly used to assess the body's ability to handle a glucose load (Pierzynowska et al. 2024). As shown in Figure 4B, after oral glucose administration, blood glucose levels peaked at 0.5 h and gradually decreased, returning to baseline by 2 h. In Figure 4C, the area under the curve (AUC) for glucose was significantly elevated (approximately 69.42%, *p* < 0.01) in the DM group compared to the NC group. However, both the medium‐ and high‐dose treatment groups showed a significant reduction in AUC, suggesting that TMP and Se‐TMP enhanced glucose metabolism, leading to lower blood glucose concentrations.

After the mice were gavaged with olive oil, the triglyceride (TG) levels in each group followed a similar trend (Figure 4D). TG levels peaked at 2 h, then began to decline, nearly returning to baseline by 4 h. The area under the curve (AUC) of the oral fat tolerance test (OFTT) was significantly lower in the intervention groups compared to the diabetic (DM) group (*p* < 0.01) (Figure 4E). These results suggest that TMP and Se‐TMP effectively reduce lipid absorption and inhibit pancreatic lipase activity, both of which are crucial in the development of type 2 diabetes and its complications. After treatment with TMP and Se‐TMP, the AUC of the OFTT in diabetic mice was significantly reduced, indicating that these treatments enhance fat metabolism and may help alleviate lipid metabolism disorders in diabetic mice.

Insulin is the primary hormone regulating blood glucose levels, and diabetes mellitus is a metabolic disorder characterized by hyperglycemia and defects in lipid and protein metabolism, often due to impaired insulin secretion or action (Falana et al. 2024). Under normal physiological circumstances, a precisely regulated equilibrium exists between the body's blood glucose levels and insulin secretion, which is crucial for maintaining normal glucose metabolism. However, when this equilibrium is perturbed and insulin resistance (IR) emerges, the body is compelled to secrete increased amounts of insulin in an attempt to sustain blood glucose homeostasis. Therefore, promoting insulin secretion and improving insulin sensitivity are key strategies in managing type 2 diabetes. Some studies have shown that extracted a polysaccharide from *Lonicera japonica* flower buds and found it increased fat index levels and improved insulin resistance in diabetic rats (Qian et al. 2023). The total polysaccharide and the neutral polysaccharide fractions isolated from *Gomphidiaceae rutilus* enhance lipolysis and autophagy to inhibit lipid accumulation in the liver, resulting in increased insulin sensitivity and thus leading to lower blood glucose (Yang et al. 2019).

Studies on termite fungus comb polysaccharides (TFCPs) have demonstrated that administration of TFCPs to diabetic mice leads to reduced serum insulin levels and marked improvement in insulin resistance. Additionally, the pancreatic islets of these mice showed more regular morphology, distinct margins, and no obvious pathological changes (Xiao et al. 2024). Separately, a study identified that selenium demonstrated insulin‐like activities when administered in rats stimulating glucose transport and insulin‐sensitive cyclic adenosine monophosphate phosphodiesterase (cAMP‐PDE) when incubated with rat adipocytes (Ezaki 1990). In our study, compared to the NC group, the FINS in the DM group was elevated. After treatment, the FINS levels in the intervention groups were reduced, with Se‐TMP showing a stronger effect than TMP, exhibiting a dose‐dependent response (Figure 4F). This enhanced effect can be attributed to the ability of selenium when selenium polysaccharide can directly regulate the insulin signaling pathway, activate the phosphorylation of insulin receptor substrate‐1 (IRS‐1), and promote the intracellular transmission of insulin signals; under the synergistic effect of selenium and polysaccharides, it enhances the activity of antioxidant enzymes, reduces the excessive accumulation of reactive oxygen species, and protects tissues and organs, thereby lowering blood glucose (Zhou et al. 2020). TMP and Se‐TMP mitigated the elevated blood glucose levels induced by T2DM, suggesting their potential therapeutic effects by protecting pancreatic β‐cells, promoting insulin secretion from pancreatic islets, and reducing insulin resistance in diabetic mice. Notably, Se‐TMP demonstrated a stronger protective effect on β‐cells compared to TMP.

#### 3.7.2 Effects of TMP and Se‐TMP on HK, PK and Glycated Hemoglobin Activities in Diabetic Mice

**FIGURE 5:**
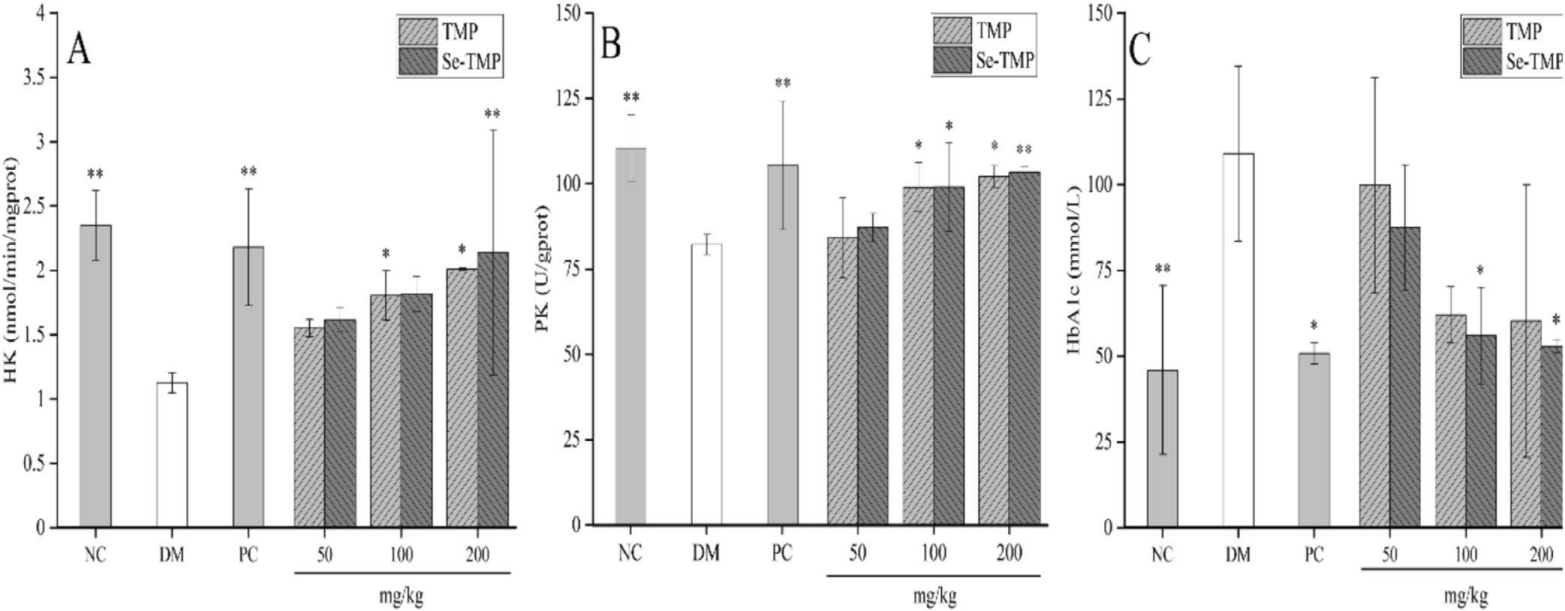
Effects of TMP and Se‐TMP on HK, PK, and glycated hemoglobin activities in diabetic mice. Panels (A–C) show the effects on HK, PK, and glycated hemoglobin, respectively. Values expressed as mean ± SD (*n* = 10/group). *and ** indicate *p* < 0.05 and *p* < 0.01 compared with the DM group, respectively.

The development of diabetes results in the downregulation of key enzymes involved in glycolysis, such as hexokinase (HK) and pyruvate kinase (PK), leading to reduced glucose utilization. To evaluate the effects of TMP and Se‐TMP on glucose metabolism, we measured the activities of HK and PK in the livers of treated mice. As shown in Figure 5A,B, both HK and PK activities were significantly lower in the DM group compared to the NC group (*p* < 0.01). In diabetic mice, no significant change in HK activity was observed in the low‐ and medium‐dose groups compared to the DM group. However, high‐dose TMP and Se‐TMP treatment significantly increased HK activity (*p* < 0.05). Additionally, PK activity was significantly elevated in the medium and high‐dose intervention groups relative to the DM group (*p* < 0.05), with a dose‐dependent response. These results suggest that TMP and Se‐TMP effectively promote glycolysis and glucose metabolism. Similar findings were reported in a previous study, where *Lycium barbarum* polysaccharides (LBP‐s‐1) enhanced HK and PK activities in the livers of diabetic animals, demonstrating a notable hypoglycemic effect (Zhu et al. 2013). Therefore, the high doses of TMP and Se‐TMP may have contributed to the improvement of glycogen synthesis and the reduction of blood glucose levels through increased liver HK and PK activities.

Glycated hemoglobin (HbA1c) reflects the non‐enzymatic binding of glucose to hemoglobin in erythrocytes and serves as a key indicator for assessing long‐term blood glucose control in diabetes. As shown in Figure 5C, HbA1c levels were significantly higher in the DM group compared to the NC group. Treatment with medium‐ and high‐dose TMP and Se‐TMP significantly reduced HbA1c levels relative to the DM group (*p* < 0.05). HbA1c is an important marker of long‐term blood glucose fluctuations. Glucose enters erythrocytes via the GLUT1 transporter, where it binds to hemoglobin to form glycated hemoglobin. GLUT1 is widely expressed in tissues such as the kidneys, nerves, and retina, which explains the strong link between diabetes and complications like nephropathy, neuropathy, and retinopathy (Leon et al. 2019). Previous studies have shown that *Astragalus* polysaccharides inhibit glucose reabsorption in renal tubular epithelial cells and reduce HbA1c levels in diabetic mice (Meng et al. 2020). Additionally, an aqueous extract of *Scrophularia ningpoensis* has been reported to lower both HbA1c and blood glucose levels in STZ‐induced diabetic rats (Yong et al. 2017). These findings suggest that TMP and Se‐TMP have beneficial regulatory and protective effects on glycated hemoglobin, potentially reducing its levels and offering protective benefits against diabetes and its complications.

#### 3.7.3 Effects of TMP and Se‐TMP on Blood Lipid Levels in Diabetic Mice

**FIGURE 6:**
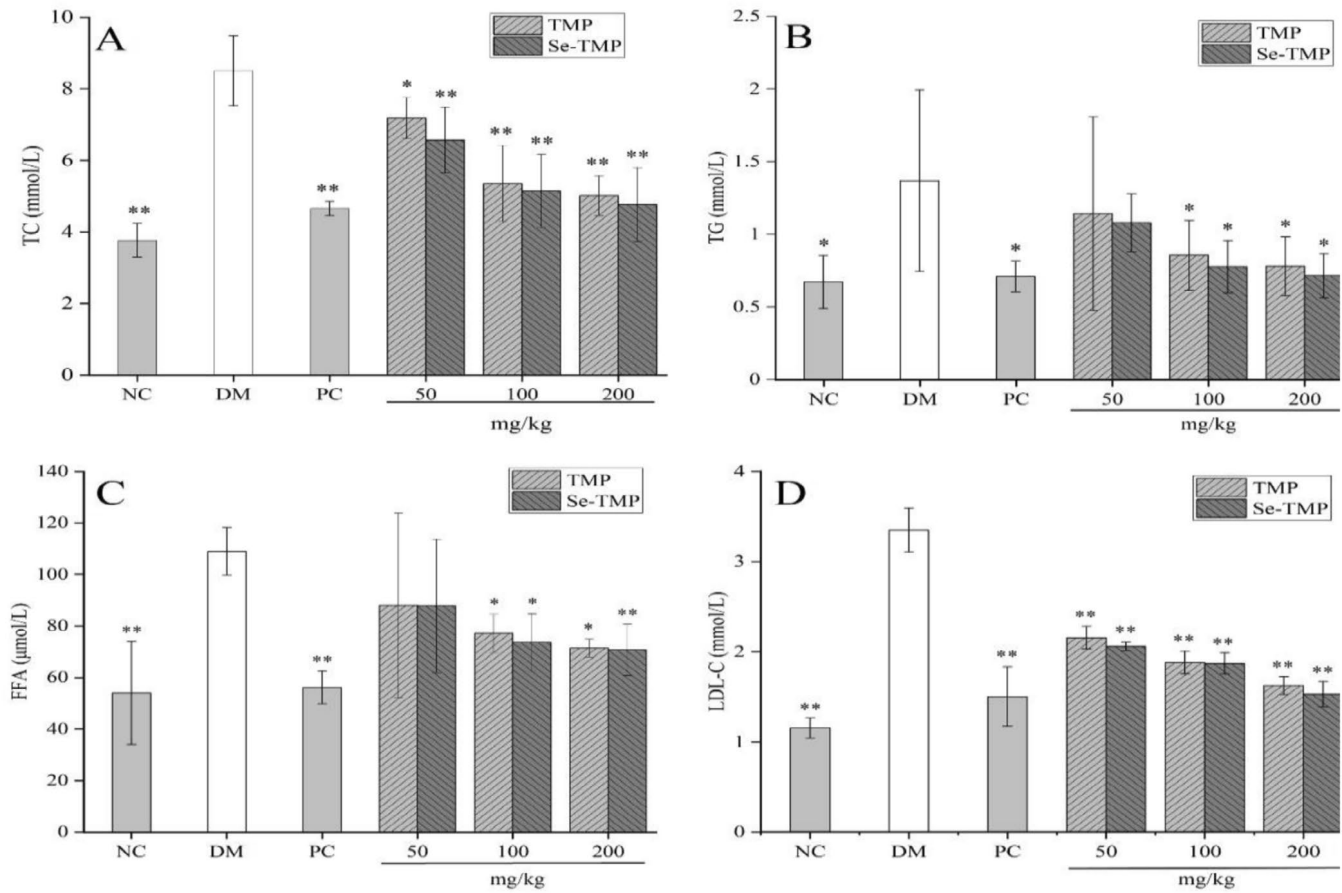
Effect of TMP and Se‐TMP on lipid levels in diabetic mice. Panels (A–E) show the effects of TMP and Se‐TMP on TC, TG, FFA and LDL‐C levels, respectively. Values expressed as mean ± SD (*n* = 10/group). * and ** indicate *p* < 0.05 and *p* < 0.01, compared to the DM group, respectively.

Diabetes is closely associated with lipid metabolism disorders, which can lead to complications such as cardiovascular disease and atherosclerosis (Wong and Sattar 2023). Therefore, improving lipid metabolism is crucial for managing type 2 diabetes mellitus (T2DM). The effects of TMP and Se‐TMP on blood lipid profiles in T2DM mice are shown in Figure 6. The levels of total cholesterol (TC), triglycerides (TG), low‐density lipoprotein cholesterol (LDL‐C), and free fatty acids (FFA) in the DM group were significantly higher compared to the NC group. In the treatment groups, TC and LDL‐C levels were significantly lower than in the DM group (*p* < 0.01), showing a dose‐dependent reduction. TG and FFA levels were significantly reduced in the medium and high‐dose TMP and Se‐TMP groups (*p* < 0.05).

In this study, TMP and Se‐TMP improved the lipid profiles of T2DM mice, with Se‐TMP showing a more pronounced effect. Some studies have found that oral administration of *Grifola frondosa* polysaccharide significantly reduced TG and LDL‐C levels, improving dyslipidemia in T2DM mice (Guo et al. 2020). Similarly, the isolated *Inonotus obliquus* polysaccharide (IOP‐A2) reduced lipid levels by promoting cholesterol metabolism and regulating key proteins such as CYP7A1, LXRα, SR‐B1, and ABCA1 (Ding et al. 2024); *Sargassum fusiforme* polysaccharide decreased the content of total cholesterol and total triglyceride in the high‐fat cells (Tang et al. 2023). TMP and Se‐TMP may lower TG, TC, FFA, and LDL‐C levels in diabetic mice, potentially through β‐cell protection or enhanced insulin secretion. However, the exact mechanisms remain to be further explored.

#### 3.7.4 Effect of TMP and Se‐TMP on Antioxidant Activity in Diabetic Mice In Vivo

Oxidative stress, primarily caused by an excess of free radicals, is a key risk factor for the onset and progression of type 2 diabetes mellitus (T2DM). Common markers of oxidative stress include malondialdehyde (MDA), superoxide dismutase (SOD), glutathione (GSH), and catalase (CAT) (Ganesan and Xu 2018). In this study, the effects of TMP and Se‐TMP were investigated on oxidative stress in both serum and liver tissues of T2DM mice, as shown in Table 2. MDA levels were significantly higher in both serum and liver samples from the diabetic (DM) group compared to the normal control (NC) group (*p* < 0.01). Treatment with TMP and Se‐TMP significantly reduced MDA levels in a dose‐dependent manner (*p* < 0.01). Both TMP and Se‐TMP also significantly increased the activities of CAT, SOD, total antioxidant capacity (TAC), and glutathione peroxidase (GSH‐Px) in the diabetic mice (*p* < 0.05, *p* < 0.01). Notably, the activities of CAT, SOD, TAC, and GSH‐Px were almost fully restored to normal (NC group) levels in the high‐dose TMP (TMP‐H) and high‐dose Se‐TMP (Se‐TMP‐H) groups. In serum, high doses of TMP and Se‐TMP significantly increased TAC activity (*p* < 0.05), and all treatment groups showed significant increases in SOD and GSH‐Px activities (*p* < 0.05). In the liver, except for the low‐dose group, treatment with TMP and Se‐TMP significantly enhanced SOD, CAT, and TAC activities (*p* < 0.01), while MDA levels were significantly reduced (*p* < 0.01). High doses of TMP and Se‐TMP also significantly increased liver GSH‐Px activity compared to the DM group (*p* < 0.01).

**TABLE 2:** Effect of TMP and Se‐TMP on serum and liver antioxidant activity in diabetic mice.

Serum						
Groups	Dosages (mg/Kg)	SOD (U/mL)	CAT (U/mL)	TAC (U/mL)	MDA (nmol/mL)	GSH‐Px (U/mL)
NC	—	306.55 ± 2.48**	191.73 ± 27.38**	7.06 ± 0.43*	3.22 ± 0.66**	382.89 ± 23.29**
DM	—	238.08 ± 27.54	109.68 ± 9.52	4.62 ± 0.21	5.9 ± 0.15	224.56 ± 16.08
PC	200	302.24 ± 17.11**	187.67 ± 5.11**	7.06 ± 0.56*	3.67 ± 0.20**	382.46 ± 65.81**
Se‐TMP‐L	50	274.27 ± 14.14*	131.96 ± 7.20	5.1 ± 0.82	4.15 ± 0.58**	302.63 ± 18.61*
TMP‐L	50	259.56 ± 39.91	129.7 ± 7.70	5.24 ± 2.88	4.44 ± 0.11*	297.37 ± 3.72*
Se‐TMP‐M	100	293.61 ± 6.90**	147.02 ± 26.54**	6.25 ± 0.19	3.71 ± 0.91**	310.53 ± 13.93**
TMP‐M	100	289.61 ± 14.90**	138.13 ± 3.20*	6.12 ± 2.49	3.78 ± 0.40**	319.3 ± 18.48**
Se‐TMP‐H	200	296 ± 22.26**	172.01 ± 5.65**	6.88 ± 0.51*	3.49 ± 1.33**	370.18 ± 31.72**
TMP‐H	200	295.36 ± 9.84**	161.32 ± 12.98**	6.57 ± 0.40*	3.79 ± 0.24**	340.35 ± 19.93**

Liver						
		U/mgprot	U/mgprot	U/mgprot	nmol/mgprot	U/mgprot
NC	—	156.12 ± 10.04**	62.06 ± 8.03**	1.96 ± 0.05**	0.81 ± 0.10**	1475.34 ± 36.68**
DM	—	126.74 ± 3.15	40.34 ± 0.52	0.94 ± 0.11	1.59 ± 0.13	992.23 ± 166.23
PC	200	149.04 ± 8.14*	57.52 ± 6.76**	1.94 ± 0.11**	0.89 ± 0.30**	1419.77 ± 123.78*
Se‐TMP‐L	50	130.98 ± 20.33	44.96 ± 1.44	1.4 ± 0.18	1.13 ± 0.10**	1080.39 ± 160.87
TMP‐L	50	128.66 ± 3.18	44.29 ± 0.95**	1.4 ± 0.32	1.12 ± 0.08**	1152.81 ± 293.94
Se‐TMP‐M	100	148.48 ± 2.68**	48.8 ± 1.89**	1.78 ± 0.34**	0.93 ± 0.21**	1301.35 ± 193.41
TMP‐M	100	144.95 ± 16.35*	47.47 ± 4.64**	1.71 ± 0.68**	0.96 ± 0.17**	1279.1 ± 148.75
Se‐TMP‐H	200	154.99 ± 7.71**	52.52 ± 1.66**	1.92 ± 0.40**	0.88 ± 0.08**	1460.15 ± 232.36**
TMP‐H	200	152.92 ± 9.25**	52.33 ± 5.53**	1.89 ± 0.34**	0.87 ± 0.12**	1402.89 ± 308.35*

*Note:* Values expressed as mean ± SD (*n* = 10/group). * and **indicate *p* < 0.05 and *p* < 0.01 compared with DM group, respectively.

Several studies have linked oxidative stress to insulin resistance, and active polysaccharides have been shown to mitigate T2DM by reducing oxidative stress (Jiang et al. 2015). For example, crude polysaccharide extracts from Chinese wolfberry have been found to prevent hyperglycemia‐induced oxidative stress in streptozotocin‐induced diabetic rats (He et al. 2019); *pumpkin* polysaccharides can lower blood glucose by reducing oxidative stress (Yang et al. 2024); *Ganoderma atrum* polysaccharides demonstrated remarkable antioxidant capacity by reversing Cd‐induced depletion of catalase, glutathione, and superoxide dismutase, while significantly reducing malondialdehyde content (Wang et al. 2025). In this study, we observed that TMP and Se‐TMP significantly increased the activities of liver SOD, CAT, T‐AOC, and GSH‐Px, while reducing MDA levels, indicating that these treatments alleviate hepatic oxidative damage and reduce oxidative stress. This may improve blood glucose control by enhancing antioxidant capacity, with Se‐TMP showing a more pronounced effect. Upon the formation of selenium polysaccharide via covalent bonding, the polysaccharide component functions as a targeted delivery vehicle for selenium, promoting its precise localization to cells involved in the insulin signaling pathway. In turn, selenium potentiates the ability of polysaccharides to modulate this pathway (Chun et al. 2016; Wang et al. 2012). As a result of this synergistic interaction, selenium polysaccharides elicit more pronounced improvements in insulin resistance and blood glucose reduction than either selenium or polysaccharides alone. Other studies have also shown that TMP can reduce cellular damage by enhancing both oxidative and antioxidant capacity in T2DM mice (Yang et al. 2021), thereby alleviating oxidative stress and ultimately lowering blood glucose.

## 4 Conclusion

In this study, we successfully developed a submerged fermentation method using *T. matsutake* mushrooms as a carrier and sodium selenite as a selenium source to produce selenium‐enriched *T. matsutake* mycelium and its polysaccharides. In vitro assays demonstrated that the antioxidant and hypoglycemic activities of Se‐TMP were both significantly higher than those of TMP. These findings suggest that selenium enrichment may be a key factor contributing to the enhanced antioxidant and hypoglycemic effects of Se‐TMP. In conclusion, selenium‐enriched submerged fermentation offers an effective approach for producing selenium‐enriched *T. matsutake* mycelium, and the resulting mycelial polysaccharides hold potential for further development as natural antioxidants and therapeutic agents for the prevention or treatment of diabetes and related diseases.

## Author Contributions

Conceptualization, X.L., N.J. and XQ.Z.; methodology, X.L., N.J., XQ.Z., Y.W. and J.L.; formal analysis, X.L., N.J., and Y.W.; investigation, J.L., Y.W., Z.Z., M.Y., XC.Z., H.L., Y.X., M.X. and Z.Y.; data curation, X.L., N.J., J.L., and Y.W.; writing – original draft preparation, J.L. and Y.W.; writing – review and editing, X.L., N.J. and J.L.; all authors have read and agreed to the published version of the manuscript.

## Ethics Statement

All the experiments were conducted according to the Guidelines of Experimental Animal Administration austerely published by the State Committee of Science and Technology of the People's Republic of China. The study was reviewed and approved by the Laboratory Animal Ethics Committee of Hubei Minzu University (No. 202043).

## Conflicts of Interest

The authors declare no conflicts of interest.

## Data Availability

The data that support the findings of this study are available from the corresponding author upon reasonable request.
